# Ecological Correlates of Group-Size Variation in a Resource-Defense Ungulate, the Sedentary Guanaco

**DOI:** 10.1371/journal.pone.0089060

**Published:** 2014-02-19

**Authors:** Andrea Marino, Ricardo Baldi

**Affiliations:** 1 UI Ecología Terrestre, Centro Nacional Patagónico, Consejo Nacional de Investigaciones Científicas y Técnicas, Puerto Madryn, Argentina; 2 Patagonian and Andean Steppe Program, Wildlife Conservation Society, CABA, Argentina; Institut Pluridisciplinaire Hubert Curien, France

## Abstract

For large herbivores, predation-risk, habitat structure and population density are often reported as major determinants of group size variation within and between species. However, whether the underlying causes of these relationships imply an ecological adaptation or are the result of a purely mechanistic process in which fusion and fragmentation events only depend on the rate of group meeting, is still under debate. The aim of this study was to model guanaco family and bachelor group sizes in contrasting ecological settings in order to test hypotheses regarding the adaptive significance of group-size variation. We surveyed guanaco group sizes within three wildlife reserves located in eastern Patagonia where guanacos occupy a mosaic of grasslands and shrublands. Two of these reserves have been free from predators for decades while in the third, pumas often prey on guanacos. All locations have experienced important changes in guanaco abundance throughout the study offering the opportunity to test for density effects. We found that bachelor group size increased with increasing density, as expected by the mechanistic approach, but was independent of habitat structure or predation risk. In contrast, the smaller and territorial family groups were larger in the predator-exposed than in the predator-free locations, and were larger in open grasslands than in shrublands. However, the influence of population density on these social units was very weak. Therefore, family group data supported the adaptive significance of group-size variation but did not support the mechanistic idea. Yet, the magnitude of the effects was small and between-population variation in family group size after controlling for habitat and predation was negligible, suggesting that plasticity of these social units is considerably low. Our results showed that different social units might respond differentially to local ecological conditions, supporting two contrasting hypotheses in a single species, and highlight the importance of taking into account the proximate interests and constraints to which group members may be exposed to when deriving predictions about group-size variation.

## Introduction

The ecological determinants of ungulate grouping patterns have intrigued ecologists for decades. Several factors, such as predation risk, habitat structure and population density are often linked to group size variation between and within species [1], [2]. However, whether these relationships result from a biological adaptation or from a purely mechanistic process with no adaptive significance, is still subject to debate [3], [4]. Regarding the adaptive approach to group-size variation, anti-predator benefits and competition costs are among the most cited arguments. Grouped prey can benefit from increased detection of predators at a safe distance [5], [6], decreased individual probability of death per encounter with a predator -dilution effect [2], and cooperative defense [7]. Anti-predator benefits increase with group size for many prey species [2] and have been considered as the main ecological factors promoting group formation in open-dwelling ungulates [8]. Jarman (1974), in his study of African antelope social organization, suggested that anti-predator benefits would impose the lower limit to group size. However, aggregation costs are expected to place the upper limit on the number of individuals that can live together [2]. In this case, Jarman (1974) proposed that, as browsers feed on single plant parts, they remove whole items thus increasing their dispersion. In contrast, grazers gradually reduce the size of food items by repeated bites but resulting in the same spread pattern from the pre-grazing condition. Therefore, grazers moving over already eaten pasture can maintain the same spacing as upon virgin pasture, and still present each individual with the same dispersion of food items, whereas browsers are forced progressively further apart as food items are removed from their original dispersion. The author suggested that as consequence, species feeding on grasses would be able to form larger groups than those of browsers.

Assuming that selection will favor individuals that optimally balance the costs and benefits of anti-predator strategies [9], we might expect group size to increase as the frequency of encounters with predators intensifies [2]. However, group size should rise until individual costs derived from grouping offset anti-predator benefits. Thus, under Jarman's hypotheses, predation risk would make group size grow until it reaches the limit placed by food dispersion, having grazers a greater potential to form large groups than browsers. Indeed, various ungulate species show greater group sizes when foraging in open habitats than when they are observed in closer habitats, such as shrublands or woodlands [4], [10], [11], [12]. But, while Jarman's theory implies that group size is an adaptive response to ecological conditions, others [3], [13] suggested that groups of large herbivores are non-permanent units and that group size increase with habitat openness is the result of a purely mechanistic process. Gerard (2002) stated that any increase in the distance at which animals perceive one another, or in population density, might increase the rate of group fusion and thus group size. Thus, the author proposed that observed group sizes are an emergent property rather than an adaptive response encoded in the individual. Empirical evidence on roe deer *(Capreolus capreolus*) supported this hypothesis [3], [4]. Finally, behavioral factors, such as territoriality, might indirectly influence group size through their effects on home-range size [14]. Defense costs are expected to increase with territory size and, as shown by many ungulate species and populations, undefended home ranges are usually larger than defended ones [14]. As this restriction is expected to limit the number of individuals that can efficiently forage together, territorial groups should be smaller than non-territorial ones.

Even though grouping patterns have been a central issue in ungulate ecology, there is a lack of comparative studies among different populations of the same species accounting for predation [10], habitat structure and population density effects. This is probably because few species exhibit such a wide geographic range to encompass the required contrasting settings [15], [16]. One exception is the guanaco *(Lama guancioe*), one of the two species of South American wild camelids, which inhabits deserts and semi deserts from Northern Perú to Tierra del Fuego. Guanaco breeding system is a resource-defense polygyny in which the main social units are families (harems) and bachelor groups [17], [18]. Their populations can be migratory (i.e. family groups gather into mixed groups after the reproductive season and move together to a winter range) or sedentary (i.e. each family group remain in the territory defended by the harem male all year round) [17], [18]. Guanaco natural predator is the puma (*Puma concolor*) which, because of its tendency to prey on livestock, by the middle of the 20th century was extirpated from most of its former range across Patagonia [19]. Although during the last twenty years hunting pressure on native carnivores was reduced and pumas have recolonized much of their former range throughout Patagonia [20], there are still some places where non-human predators of guanacos have been absent for decades, offering the unusual opportunity to test predation-risk hypotheses.

Guanaco group size varies within and between populations [21] but until now there were no comparative studies accounting for ecological factors responsible for those differences. The aim of this study was to model guanaco group size in contrasting scenarios of predation risk, habitat structure and population density, under the current theoretical framework. Our main predictions were that 1) group size would be larger at sites where predation risk is high than at predator-free sites, 2) guanacos would form larger groups in grasslands than in shrublands, and 3) group size would increase with population density. In addition, we expected that, because male capability to effectively defend a territory should constrain territory size and the number of females in it, 4) family groups to be smaller than bachelor groups. As costs/benefits perception is expected to differ between individuals living in family and bachelor groups, we tested predictions 1–3 separately for each of these social units.

## Study Locations

In order to compare group sizes of guanacos in contrasting scenarios of habitat structure and predation risk, we conducted population surveys in three protected areas of eastern Patagonia, where poaching does not occur due to the presence of permanent wardens. All of these areas have experienced important changes in guanaco density throughout the study period.

San Pablo de Valdés (SP) is a 7,300 has ranch located in the Southern portion of Península Valdés, in Chubut Province, Argentina (42°36′S; 64°15′W). The most distinctive climatic factor across the peninsula is the low average annual rainfall (280 mm), which falls mostly in the autumn and winter. A detailed description of vegetation communities can be found in Burgi et. al. [22] but on a general basis, it is composed by shrublands and grasslands typical of the Patagonian Province [23]. Formerly dedicated to sheep production, SP was purchased in 2005 by a local NGO to be turned into a wildlife reserve; all the c. 3,500 sheep were removed and a permanent warden appointed. Since then, guanaco population increased systematically [22]. Pumas are reported only occasionally inside Península Valdés (Marcela Nabte, personal communication) and predation risk at SP during this study can be considered null.

Cabo Dos Bahias (C2B) is a small wildlife reserve (1,700 ha) located in southeastern Chubut (44°55′S; 65°31′W) that holds the densest guanaco population reported. Average annual precipitation is 250 mm [24]. The vegetation in this area is characteristic of the Patagonian Province and composed of shrublands and grasslands. Shrublands are characterized by *Chuquiraga avellanedae* and *Lycium chilense*, and grasslands by *Stipa tenuis* and *Poa ligularis* (Beeskow et al. 1987). There have been no reports of guanaco predators in the area for more than 20 years (Provincial wardens, personal communication).

La Esperanza (LE) is a privately owned 6700 ha protected area located in Chubut Province, (42°7′; 64°57′W). Average annual rainfall is 200 mm. The vegetation is characteristic of the Southern Monte but shares plant species with the Patagonian Province in the coastal area [25]. The creosote bushes *Larrea nitida* and *L. Divaricata* dominates the western side of the ranch where the Monte prevails across the higher plains, whereas the quilimbay (*Chuquiraga avellanedae*) dominate the cliffs and canyons towards the coastal steppe. The most abundant grasses are *Stipa tenuis* and *Poa ligulari*s [26]. LE is located less than 80 kilometers North-Westward from SP but in this area puma predation on guanaco and sheep is common, and guanaco individual behavior is consistent with high predation-risk level [27].

Guanaco diets at SP and C2B are similar, with *Poa sp.*, *Stipa sp.* and *Chuquiraga sp.* as main items (Marino, unpublished data). Presumably, guanaco diet at LE is composed by the same preferred items since edible plants reported at the three reserves are almost the same. The other two species of medium-sized herbivores occurring in these locations are choiques (*Rhea pennata pennata*) and maras (*Dolichotis patagonum*), both of them at extremely low densities. Domestic sheep (*Ovis aries*) may enter occasionally into the reserves from neighboring ranches but are rapidly removed by wardens or ranchers. Hence, we assumed that there is no interspecific competition affecting guanacos in our study locations. Telemetry studies conducted at LE [28], and the lack of seasonal changes in family-groups density across more than eight years of surveys (Marino, unpublished data) indicate that guanacos are sedentary within these reserves.

## Methods

### Field Methods

We conducted 25 population surveys. Annual, post-reproductive (December–March) surveys were conducted in 2006, and 2008–2012 period at SP; in 2006–2012 period at C2B; and 2008–2011 period at LE. We also conducted pre-reproductive surveys (September–October) in 2006–2008 and 2010 at SP; 2006–2008 at C2B; and 2007, 2009 and 2012 at LE. Field work was authorized by the Dirección General de Conservación de Áreas Protegidas, SubsecretarÍa de Turismo y Áreas Protegidas, and the Dirección de Fauna y Flora de la Provincia de Chubut (Exp. N° 004108-MCETI/10; Res. N° 052/05; Disp. N°033/2011-DFyFS-SSRN, Disp. N° 48/2008), which are the relevant regulatory bodies concerned with protection of wildlife and animal ethics issues within public and private protected areas from Chubut province. Additionally, within both private reserves, surveys were conducted with the personal supervision and collaboration of the wardens and administrators in charge, who often include them as training activities for transient volunteers. This study did not involve endangered species or any kind of animal handling, and it was purely observational. These surveys, that were oriented to estimate population density and social organization variables, were conducted between 9:00 a.m. and 8 p.m. Data were collected by two observers standing in the back of an open pick-up vehicle. Whenever a group of guanacos was detected, we stopped the vehicle and counted the individuals, trying to identify age and sex categories with 40× binoculars. Observed groups were classified into three social categories. Family groups (1) were composed by an adult male and one or more females, with or without offspring. Guanacos younger than six months and yearlings can be recognized easily but adult females and adult males are very similar. Gender of adult guanacos was determined by direct observation of external sexual organs and/or lactating young. In addition to group composition, behavioral patterns, which are well studied in these locations [27], [29], [30], [31] were considered to assign groups to this category: harem males tend to be some meters away from the entire group, often showing some degree aggressive and/or territorial displays towards group members and neighboring groups, as chases and defecating. Females in family groups tend to be highly cohesive, with a high a degree of synchronization in their activities. In contrast, bachelor groups (2) are composed mostly by juvenile and adult males. These groups lack from cohesion or clear hierarchies, and are loosely aggregated, with individuals entering and leaving continually [30]. Finally, we considered a group as *undetermined* (3) when it was too far away for us to assign sex categories properly and/or behavioral interactions were not clear enough. Solitary individuals were not considered in this analysis and they represented on average 3% (±1.6) of the observed individuals in each population. We used the distance between individuals as accessory data to define a group size only for bachelor groups because as harem males tend to chase intrudes for long distances and territorial tolerance vary between populations, this measure can be misleading for family groups. We considered an individual as a part of a bachelor group if it was less than 200 meters away from the rest. In order to test our predictions, we defined group size as the number of individuals older than 12 months. We did not considered young individuals (younger than one year old) because their number is a direct consequence of the number of females, they are significantly smaller than adults and consume less forage to assume the same level of competition of an adult, they do not contribute to predator detection, and they suffer greater mortality so the number of young in a group can vary markedly in the short term [29], [32]. Therefore we expected our predictions to work with older individuals, and each juvenile was considered with its mother as one individual with high energetic demands.

We also registered the distance between the group and the vehicle, group and road trail azimuth, geographic location (GPS) and finally, a general visual description of the vegetation patch where the group was located, considering as a patch an area of 200–300 m around the group. Patch description was classified into vegetation types considering dominant functional groups and dominant plant species (see *Habitat structure and forage availability subsection*).

### Habitat structure and forage availability

Habitat structure at patch scale.- Vegetation data gathered during the surveys were grouped into two categories: (1) grasslands, dominated by grasses, with less than 10% of shrub cover; and (2) shurblands, with shrubs covering more than 10% of soil surface. An intermediate level, with shrubs covering between 10 and 20% of soil surface, was explored but subsequently removed due to (a) the difficulties of assessing shrub cover level accurately from the vehicle and (b) the lack of significant differences in size between groups observed in patches with high and intermediate shrub cover. Therefore, data with variable shrub cover higher than 10% were pooled into a single category “shrublands”. Converting habitat structure in a two-level categorical variable has been a useful approach to explain the observed variation in individual behavior between contrasting predation-risk settings [29], supporting the use of this variable to study social responses to predation-risk.

To assess the influence of habitat type at landscape scale, we incorporated information about vegetation communities into a Geographic Information System. We used available vegetation maps assembled from aerial photographs and classified images [23], [33], [34]. Vehicle locations (Global Positioning System), azimuth and distance (Laser rangefinder Bushnell 1000) to the group, allowed us to calculate the exact location of the group and assign a vegetation stratum to each group observed.

Enhanced Vegetation Index (EVI) derived from MODIS satellite images of 250 m of resolution was used as a measure of relative forage availability [35]. These data are distributed by the Land Processes Distributed Active Archive Center (LP DAAC) (lpdaac.usgs.gov). We assigned the corresponding pixel value to each group observed as an indicator of green biomass at a patch scale.

### Local density estimation

In order to account for group size differences due to local density variations, we used distance data to estimate local abundance through *Distance Sampling* [36], using *Distance 5.0* software.

### Statistical analysis

To conduct the comparative analysis on group size we fitted general linear mixed models with a logarithmic link function and a Poisson error distribution, which is a combination usually recommended to model count data [37]. We first fitted a global model to compare family groups with bachelor groups. Afterwards, we fitted separate models to family group and bachelor group data to test the rest of our predictions. In order to account for variation inherent to each site, we included Site as a random factor. To test our predictions, we considered predation (high vs null predation risk level), habitat structure at patch scale (grasslands vs shrublands), vegetation stratum at landscape scale and population density, as fixed effects. Density range differed so much among locations that it was impossible to include raw data into our models. Therefore, in order to simultaneously account for density effect on group size within each reserve, density data from each site was centered by subtracting the site average. In addition, we included green index EVI at patch scale (pixel value) in our models to account for potential effects of local primary productivity on group-size variation. Finally, to account for intra-annual temporal variation on group size, we classified our surveys into (1) pre-reproductive (i.e. before November 1^st^) and (2) post-reproductive (i.e. after November 1^st^). Bachelor group sizes were over-dispersed (the residual deviance was eight times the residual degrees of freedom) therefore, we fitted a negative binomial model to this data set [38]. At the moment of this study, software packages for fitting negative binomial mixed models were under developing or testing stages. Therefore, in order to confirm that there were no differences among reserves after controlling for the other variables, we compared the AIC score of our final model with the AIC score of the same model including the factor Site as an additional fixed effect instead of including it as a random term. Model selection was based on Akaike Information Criteria (AIC) [39]. We first selected a set of models based on a delta AIC<2 respect from the model having the lowest AIC [40]. Among these candidates, we considered the most parsimonious model the simplest alternative (i.e. less parameters) [40]. If the candidates had the same number of parameters, we chose the one with lowest AIC. Although we used an information approach to model selection, we present parameter estimates of those factors included in the final models, with their corresponding standard errors and p values, in order to describe effect sizes and precision matters. Model fitting was performed using Lme4 and nlme packages for R software (version 2.15.2, The R Foundation for Statistical Computing, www.r-project.org).

## Results

### Descriptive statistics

**Figure 1:**
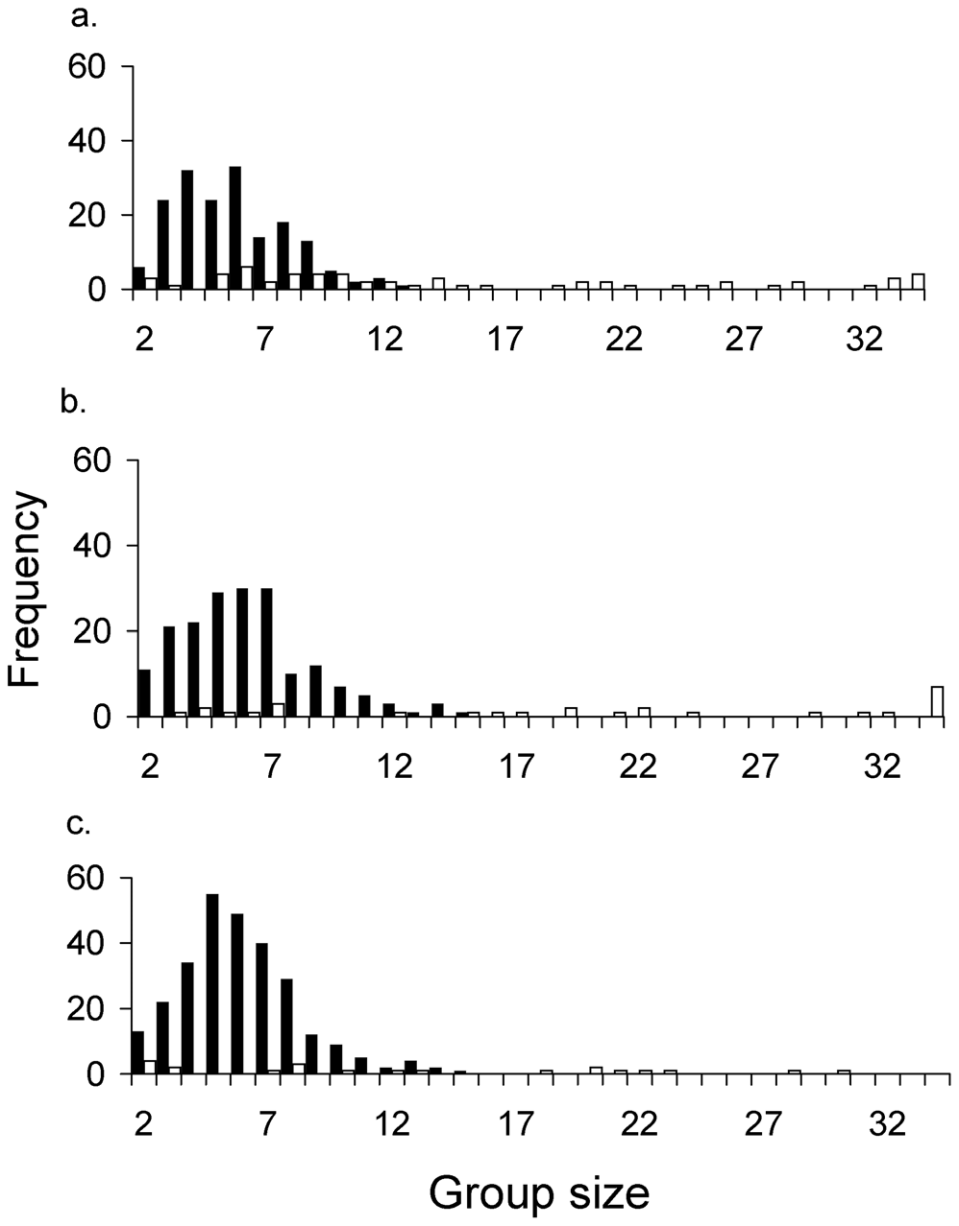
Group-size distribution at a) Cabo Dos Bahías (C2B), (b) San Pablo (SP), and c) at the predator-exposed site La Esperanza (LE) (data pooled across years). Dark and light bars represent family and bachelor groups respectively.

On average, family groups were composed of 6 adult individuals, ranging from pairs to an adult male with 14 females (Table 1). Although such a large group was very uncommon, only 5% of the 637 family groups surveyed were larger than ten individuals. In fact, 64% of the observations comprised six individuals or less. Average and median family size were consistent across the three populations sampled (Table 1). Family group size showed the typical Poisson-like frequency distribution, moderately skewed to the right (Figure 1, Table 1). Skewness and kurtosis are useful metrics to explore frequency distribution shape. Skewness level measures the extent to which a distribution has long, drawn-out tails on one side or the other, relative to a normal distribution (which is symmetrical) [38]. Negative values mean skew to the left (negative skew) and positive values mean skew to the right. Kurtosis has to do with the peakyness, or flat-toppedness, of a distribution [38]. A positive kurtosis value indicates a more pointy (i.e. leptokurtic) distribution than the normal. Group size distribution showed a sharper peak around the mean at LE (high predation-risk level) than family groups of the predator-free sites, as indicated by kurtosis coefficients (Table 1).

**Table 1:** Descriptive statistics for family and bachelor group size at the study locations, pooled across the years of study.

Group Type	Site	PR	Density	N	Mean (SD)	Median	Range	%CV	Kurtosis	Skewness
Family	SP	Null	3.95–26.3	185	6.1 (2.7)	6	2–15	44.1	0.68 (0.3)	0.82 (0.2)
Family	C2B	Null	44.8–70.1	175	5.8 (2.3)	6	2–13	40.4	−0.02 (0.4)	0.64 (0.2)
Family	LE	High	6.7–13.4	277	6.1 (2.4)	6	2–15	39.7	1.15 (0.3)	0.85 (0.1)
Bachelor	SP	Null	3.95–26.3	28	23.0 (17.2)	20	3–75	74.7	1.18 (0.9)	1.1 (0.4)
Bachelor	C2B	Null	44.8–70.1	59	15.6 (11.1)	11	2–51	71.4	0.25 (0.6)	0.97 (0.3)
Bachelor	LE	High	6.7–13.4	21	12.5 (9.2)	10	2–30	73.5	−1.13 (1.0)	0.40 (0.5)

Predation-risk level (PR), population density range expressed as guanacos.km^−2^ (Density), sample sizes (N), mean group sizes and standard deviations (SD), range of observed group sizes (Range), coefficient of variation (%CV), kurtosis (standard error of kurtosis) and skewness (standard error of skewness).

Bachelor group sizes were larger and more variable than family groups. The average bachelor group was composed of 17 individuals, ranging from pairs to 75 guanacos (Table 1) and showing great dispersion (Figure 1). Regarding frequency distribution shape, only SP and C2B showed some evidence of positive skweness (Table 1).

### Group-size correlates

**Figure 2:**
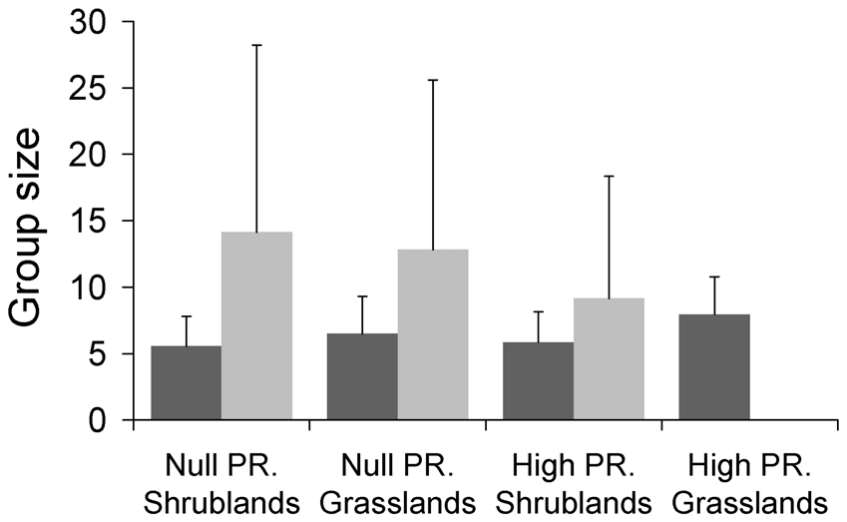
Average group size observed per combinantion of habitat structure and predation-risk (PR) level (data pooled across years). Dark and light bars represent family and bachelor groups respectively. Error bars indicate standard deviations.

**Figure 3:**
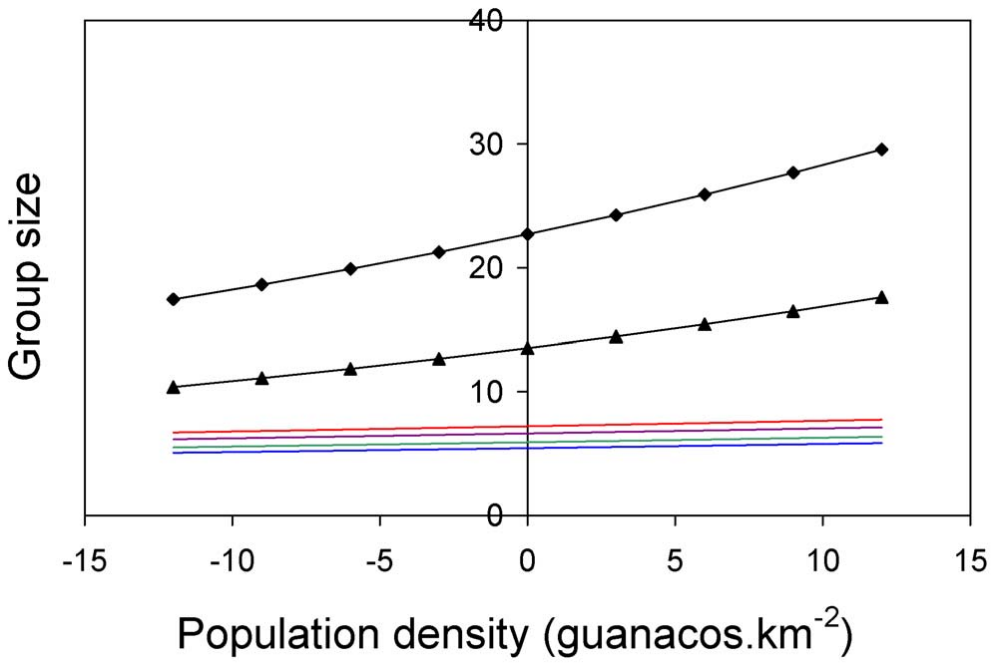
Model predictions of group size as a function of within-population variation in density (population-density data was centered by subtacting the local average to allow including the three locations in the same model). Bachelors in post-reproductive season (black line with triangle markers), bachelors in pre-reproductive season (black line with diamond markers), family groups in high predation-risk grasslands (red line), family groups in null predation-risk grasslands (purple line), family groups in high predation-risk shrublands (green line), family groups in low predation-risk shrublands (blue line).

Family groups - The minimum adequate model for family group size included the intercept, predation risk, habitat structure and population density effects (Table 2). Family groups were significantly larger in the predator exposed population than in the predator free ones (Difference = 0.084 SE = 0.035 p = 0.016, Figure 2). Additionally, groups located in grasslands were larger than those located in patches with more than 10% of shrub cover (Difference = 0.197 SE = 0.038 p<0.001, Figure 2). It is worth mentioning that predation-risk∶habitat interaction appeared among some of the best candidates (Table 2) but according to our selection criterion it had to be removed from the final model, suggesting that both effects are additive. Finally, there was a very weak but statistically significant, positive relationship between group size and population density (Slope = 0.006 SE = 0.003 P = 0.033, Figure 3).

**Table 2:** Delta AIC scores of the best models obtained for family group size within a threshold of seven AIC units and the final model selected according to our criterion (delta AIC threshold of two units).

Model	Intercept	Season	Density	Landscape	EVI	Patch	PR	Patch:PR	df	Delta AIC	weight
59	1.451		0.006296		1.953	+	+		5	0	0.157
123	1.489		0.006064		1.753	+	+	+	6	0.25	0.138
116	1.693	+	0.006144			+	+	+	6	1.18	0.087
115	1.71		0.005778			+	+	+	5	1.26	0.083
60	1.488	+	0.006475		1.58	+	+		6	1.29	0.082
52	1.678	+	0.006384			+	+		5	1.39	0.078
124	1.524	+	0.006242		1.394	+	+	+	7	1.58	0.071
Selected	1.694		0.006007			+	+		4	1.83	0.063
121	1.514				1.625	+	+	+	5	2.86	0.037
57	1.474				1.835	+	+		4	2.99	0.035
113	1.718					+	+	+	4	3.46	0.028
114	1.704	+				+	+	+	5	3.87	0.023
20	1.718	+	0.006176			+			4	4.32	0.018
49	1.702					+	+		3	4.38	0.018
122	1.543	+			1.327	+	+	+	6	4.43	0.017
50	1.688	+				+	+		4	4.47	0.017
58	1.505	+			1.525	+	+		5	4.52	0.016
19	1.74		0.005723			+			3	5.57	0.01
28	1.763	+	0.00619		−0.4401	+			5	6.01	0.008
18	1.726	+				+			3	7.22	0.004

Variables included in the full model were the Intercept, season (pre vs post-reproductive), population density, landscape stratum, EVI, vegetation structure at patch level, predation-risk level (PR), and vegetation patch-predation-risk interaction (Patch:PR). Last columns show degrees of freedom (df), delta AIC and AIC weights.

Bachelor groups - The minimum adequate model for bachelor group size included the intercept, the effect of population density and season (Table 3). We found a positive relationship between group size and density (slope = 0.022 SE = 0.01 p = 0.025, Figure 3). Regarding seasonal variation, bachelor groups were significantly larger before the start of the mating season (before November) than during late spring-summer (Diference = 0.52 SE = 0.14 p<0.001, Figure 3). Of the rest of the variables considered, EVI, habitat structure at patch scale, and predation-risk showed some influence on group size but the effects were weak and inconsistent across models. We could not test for the interaction effect between habitat type at patch scale and predation-risk level because no bachelor groups were observed in grasslands at the high predation-risk location. As expected, there were no between-population differences in bachelor group size when the other two variables were present in model (AIC_final model_ = 805.1 vs AIC_including site_ = 804.9).

**Table 3:** Delta AIC scores of the best models for bachelor group size obtained within a threshold of seven AIC units, including the final model selected according to our criterion (delta AIC threshold of two units).

Model	Intercept	Season	Density	Landscape	EVI	Patch	PR	Patch:PR	df	Delta AIC	weight
12	2.468	+	0.02374		1.192				5	0	0.169
36	2.662	+	0.02226				+		5	0.38	0.14
44	2.529	+	0.02387		1.118		+		6	0.39	0.139
Selected	2.604	+	0.02198						4	0.45	0.135
28	2.473	+	0.02376		1.194	+			6	2.22	0.056
52	2.686	+	0.02234			+	+		6	2.36	0.052
116	2.686	+	0.02234			+	+	+	6	2.36	0.052
60	2.552	+	0.02395		1.119	+	+		7	2.43	0.05
124	2.552	+	0.02395		1.119	+	+	+	7	2.43	0.05
20	2.609	+	0.022			+			5	2.63	0.045
34	2.688	+					+		4	4.27	0.02
2	2.63	+							3	4.32	0.019
10	2.522	+			0.9585				4	4.81	0.015
42	2.585	+			0.8801		+		5	5.13	0.013
50	2.712	+				+	+		5	6.22	0.008
114	2.712	+				+	+	+	5	6.22	0.008
18	2.634	+				+			4	6.46	0.007
26	2.526	+			0.96	+			5	6.99	0.005
58	2.607	+			0.8788	+	+		6	7.15	0.005
122	2.607	+			0.8788	+	+	+	6	7.15	0.005

Variables included in the full model were the Intercept, season (pre vs post-reproductive), population density, landscape stratum, EVI, vegetation structure at patch level, predation-risk level (PR), and vegetation patch-predation-risk interaction (Patch:PR). Last columns show degrees of freedom (df), delta AIC and AIC weights.

## Discussion

Our results showed that family and bachelor group-sizes were influenced by different ecological factors. Only family groups supported our hypothesis regarding group size increase in a high predation-risk setting since harem size was larger in the puma exposed population than in the predator-free reserves. Conversely, this effect was absent among bachelor groups. The fact that guanaco family group sizes supported Jarman's (1974) predictions regarding predation-risk whereas bachelor groups did not is consistent with previous findings on guanaco individual behavior. Bachelor guanacos usually show low investment in individual vigilance, regardless of group size or predation risk level [30]. In contrast, in high predation-risk settings, territorial males in low density populations and females in general, show important reductions in vigilance effort as group size increases. However, these effects are absent in predator free locations [27], [29], [30]. These differential patterns suggest that family members perceive anti-predator benefits of increasing group size whereas bachelors do not. As our results showed, bachelor groups are considerably larger than family groups and it is likely that anti-predator benefits derived from aggregation have already reached an asymptote below the average size of the former [30].

There are few studies accounting for predation-risk level to explain group-size variation within ungulate species and their results not always support the anti-predator hypothesis. Group size of zebra (*Equus burchelli*) and wildebeest (*Connochaetes taurinus*) increased with increasing predation-risk by lions (*Panthera leo*) [12] according to the expected adaptive adjustment of group size. In contrast, spatial variation in predation-risk by wolves (*Canis lupus*) had no effect on elk (*Cervus elaphus*) herd size [10]. Blackbuck (*Antilope cervicapra*) group size was also independent of predation-risk by wolves [41]. As pumas hunt through stalking, early detection conferred by a larger group could be especially advantageous for guanacos due to increased chances of escaping, as observed among Thomson's gazelles (*Gazella thomsoni*) attacked by stalking cheetahs *(Acinonyx jubatus)* [42]. But these benefits could easily decrease facing cursorial predators such as wolves, which can chase their prey for long distances, reducing early-detection advantages. As Thaker et al. (2010) suggested, the effectiveness of an anti-predator strategy would be related to the particular predator hunting pattern. These differences between predator hunting strategies may explain why group-size responses to risk level differ within and between prey species exposed to different predators.

Habitat structure had no effect on bachelor group size whereas families located in grassland patches were larger than those located in shrublands, and this effect was independent of predation risk level. Various ungulate species have shown a positive correlation between group size and habitat openness, such as elk [10], wildebeest and zebra [12], blackbuck [41], axis deer *(Axis axis)* [11] and roe deer *(Capreolus capreolus)* [4], [43]. However, very different processes have been proposed to explain the observed relationships, including the interaction between habitat structure and forage abundance, predation-risk or population density. In our study case, previous knowledge on individual behavior again predicts the observed pattern in group size variation. Between female aggression-rate increases rapidly with group size when family groups are feeding around shrubs whereas aggressive interactions are almost inexistent among group members located in grasslands [29], or among bachelors feeding in any habitat type [30]. Thus, interference competition may add to territorial-defense effort to constrain family group size more rapidly in shrublands than in grasslands, as predicted by Jarman (1974). Therefore, in our case study, individual behavior correlates and the lack of effect on bachelor groups are in accordance with the adaptive significance of the group-size increase in open grasslands.

Regarding population density, both family and bachelor groups sizes increased with guanaco local abundance. However, the magnitude of this relationship differed greatly among social-unit types. For example, while an increase of approximately three guanacos.km^−2^ would result in a one-unit increase in bachelor group size, a change in population density of almost 30 individuals.km^−2^ would be required to add one individual to the average family group. Considering that this 30-fold increase/difference in population density is very unlikely to take place in the wild, the biological sense of this relationship is questionable. Therefore, we did find a relationship between population-density and bachelor group size but, regarding family groups, density effect was so weak that prevented to draw the same conclusion. The lack of between-population variation after controlling for the fixed effects indicates that mean sizes of family groups remain constant in a wide range of population densities (4–70 guanacos.km^−2^). Evidence of a positive relationship between group size and population density was found in elk [44], axis deer [11] and white-lipped peccary (*Tayassu pecari*) [45], among many other ungulate species. Goitered gazelles *(Gazella subgutturosa)*, for example, showed a non-linear density-dependent response in which a sevenfold difference in density was needed to induce relevant group size changes [46]. In their study, Blank et al. [46] stated that density effects on group size would be more common among ungulates that tend to form large groups than those than form small groups or that are territorial. Our findings regarding within-population variation in group size are in accordance with this idea, with density-independent and relatively small territorial families, and larger bachelor groups as density increases.

In summary, our results indicate that predation risk and habitat structure influence the size of guanaco family groups whereas bachelor group size is linked to population density. Thus, family groups supported Jarman's hypotheses but the larger and more variable bachelor groups were consistent with Gerard's idea [3]. The notion of bachelor group size as an emergent property is reasonable given the fusion-fission nature of these non-territorial groups. On the other hand, in the more stable family groups, defense effort by males may restrict territory size [14] and the consequent intra-group competition costs may limit the number of females that can forage efficiently in it. In this context, the trade-off between the costs and benefits of group living for family members may impose a more dramatic constraint to family group size, and the adaptive adjustment proposed by Jarman seems an evolutionary advantageous alternative. The sharper peak around mean family group-size of the predator-exposed frequency distribution is in accordance with this idea. However, it is important to note that, though more prominent than the density effect, the magnitude of habitat and predation effects on family group size were still relatively small. The smallest family groups predicted by our model were composed of 5 and the largest of 8 members. Moreover, 50% of the family groups recorded at each location were composed of 5–7 members, regardless predation-risk level. For example, typical group size of roe deer *(Capreolus capreolus)*, a highly plastic species, consists of less than 5 individuals when they occur in woodlands but it is larger than 50 individuals when they forage in open grasslands [47], [48]. Hence, even though we found evidence supporting Jarman's ideas, this was relatively subtle in biological terms. Guanaco family groups accounted for 70–80% of the groups sampled in each population; as a result, social configuration was strongly determined by these social units. Thus, while individual behavior has proven to be highly flexible and allow guanacos to adjust to contrasting local conditions [29], social plasticity of sedentary populations seems to be relatively low. Since grouping patterns have great influence on herbivore spatial distribution and resource use, low social plasticity might have critical implications in terms of management and conservation. Future studies will help to assess the consequences of guanaco social system for other aspects of their ecology.

Ungulate species, and even populations, can differ markedly in terms of mating systems [16]and types of predators [46], and it is reasonable to expect these differences to have their correlates on social organization. Anti-predator responses may differ in front of predators with contrasting hunting strategies, and what it is advantageous facing a lone, stalking felid may entail no benefits when facing cursorial predators. To overlook these issues in the search for massive generalizations will probably lead to contradictory outcomes. Our results show that, according to the nature of the social unit considered, group-size variation can be consistent with the adaptive or the mechanistic approach, and highlight the importance of taking into account the proximate interests and constraints to which the members of the different social units may be exposed to, when deriving predictions about group-size variation.
